# Correlation of microsynteny conservation and disease gene distribution in mammalian genomes

**DOI:** 10.1186/1471-2164-10-521

**Published:** 2009-11-12

**Authors:** Simon C Lovell, Xiting Li, Nimmi R Weerasinghe, Kathryn E Hentges

**Affiliations:** 1 Hangzhou Centre for Diseases Prevention and Control, Wulin Road No.277, Hangzhou 310006 PR China; 2 College of Life Sciences, University of Dundee, Dundee, DD1 5EH, UK; 3 Faculty of Life Sciences, University of Manchester, Manchester, M13 9PT UK

## Abstract

**Background:**

With the completion of the whole genome sequence for many organisms, investigations into genomic structure have revealed that gene distribution is variable, and that genes with similar function or expression are located within clusters. This clustering suggests that there are evolutionary constraints that determine genome architecture. However, as most of the evidence for constraints on genome evolution comes from studies on yeast, it is unclear how much of this prior work can be extrapolated to mammalian genomes. Therefore, in this work we wished to examine the constraints on regions of the mammalian genome containing conserved gene clusters.

**Results:**

We first identified regions of the mouse genome with microsynteny conservation by comparing gene arrangement in the mouse genome to the human, rat, and dog genomes. We then asked if any particular gene types were found preferentially in conserved regions. We found a significant correlation between conserved microsynteny and the density of mouse orthologs of human disease genes, suggesting that disease genes are clustered in genomic regions of increased microsynteny conservation.

**Conclusion:**

The correlation between microsynteny conservation and disease gene locations indicates that regions of the mouse genome with microsynteny conservation may contain undiscovered human disease genes. This study not only demonstrates that gene function constrains mammalian genome organization, but also identifies regions of the mouse genome that can be experimentally examined to produce mouse models of human disease.

## Background

The availability of several mammalian genome sequences has enabled comparative genomic studies to identify regions of conserved linkage among different organisms (reviewed in [1-4]). These studies have been used to predict the genome architecture of the common mammalian ancestor [5,6], as well as to assess recombination [7,8] and genome evolution [9]. Additionally, regulatory elements have been identified by the characterization of conserved regions of non-coding DNA [10-12]. Non-coding functional RNAs and microRNA targets have also been identified through comparative genomic approaches [3,13,14]. Gene function may be inferred from conserved proteins in other species. Likewise, comparative genomics among mammalian species is useful for predicting the functional consequences of mutations in human disease loci [15-18]. Additionally, mapping genes responsible for quantitative traits in rodents allows the prediction of locations of human quantitative traits underlying disease, based on conserved genomic structure between rodents and humans [19-22].

Although it is becoming increasingly apparent that genomes display a large degree of structural plasticity, there are nevertheless significant evolutionary constraints on genome structure. Previous studies have provided evidence for functional constraints on genome organization in prokaryotic and eukaryotic genomes [23]. Studies in yeast demonstrate that essential genes are found in genomic clusters [24]. The clustering of essential genes in yeast is likely driven by selection for reduced noise in gene expression levels, as essential gene clusters are localized in regions of open chromatin [25]. Additionally, in the nematode *C. elegans*, essential genes are located in clusters in regions with low recombination [26].

Clustering of genes with similar functions has also been observed in mammalian genomes. In the human genome, genes that are in the same pathway are in closer proximity than would be expected by chance [27]. Similarly, in the mouse genome, genes with common GO annotations are found in clusters [28]. This is not due to tandem duplications, as most genes in the same pathway that are adjacent in the genome do not arise from duplication events [27]. It is possible that functionally related genes are located in clusters to facilitate coordinated transcription, as many genes in clusters are co-expressed [27].

Many of the previous studies to detect gene clustering were based on bioinformatic analysis of genome annotation. However, there is also support for functional constraints on mammalian genome organization from experimental data. Analysis of saturation levels of mouse mutagenesis screens for lethal phenotypes directed at specific genomic regions demonstrated that mouse essential genes are disproportionately found in regions of conserved microsynteny [29], at least for the small number of genomic regions evaluated. To build on this prior work, we assessed microsynteny conservation on all mouse autosomes. By examining gene content in conserved and divergent genomic regions we found a significant correlation between microsynteny conservation and the density of mouse genes that are orthologous to human disease genes. As the mouse is widely used to model human disease [1,30], the identification of this correlation will facilitate the creation of new mouse models of human disease by identifying regions of the mouse genome that contain a high density of disease gene orthologs.

## Results

### Microsynteny conservation of mouse autosomes

We evaluated the level of microsynteny conservation between the mouse genome and those of human, dog and rat. First, we obtained all protein-coding genes and their genomic locations on all mouse autosomes as annotated in the Ensembl mouse genome browser [3,31] (release 50). We also obtained protein-coding genes from the human [32,33], rat [2], and dog [34] genomes. These were chosen because they had a sufficient level of assembly and annotation to allow comparison. The use of the dog genome as an outgroup to human, rat, and mouse improves the stringency of the study [35]. To identify orthologs of mouse genes in the other genomes, along with their genomic locations, Ensembl BioMart homology filters were used to compile a list of orthologous genes.

Although the four mammalian genomes chosen are those with the best available annotation, the degree and quality of annotation may vary somewhat between species. In order to control for this we took additional steps to find the human, rat, and dog orthologs of mouse genes. Protein sequences of all mouse genes that did not have an annotated ortholog in another species were searched using BLAT [36] against the other genomes to identify orthologous sequences in the other genomes. To allow a moderately strict search with a limited number of false positives, all hits with E-value < 10^-5 ^were retrieved. The genomic location of the best BLAT match in the other genome was used for the evaluation of microsynteny conservation. We searched a total of 6173 genes in at least one other species, finding an ortholog for 1210 of the genes in other genomes. A sensitivity analysis demonstrates that the number of genes retrieved from the BLAT searches is relatively insensitive to the choice of E-value cut-off, as changing the cut-off point from 10^-3 ^to 10^-7 ^results in 1266 - 1169 ortholog annotations respectively. Therefore, utilizing alternate E-value cut-off points in this range would have changed the annotation of only 0.48% of the total mouse genes analyzed in our study.

Genes were defined as having conserved microsynteny if their orthologs had the same two orthologous neighboring genes in all four species examined. Each mouse gene was queried to determine whether it met these criteria for conserved microsynteny. We then assessed the level of conservation for segments of the mouse genome, determining the percentage of conserved genes in each segment. We examined 20 Mb regions of the mouse genome, as genomic regions of this size have been analyzed experimentally through region-specific mutagenesis [37-40]. Thus, the identification of additional genomic regions with conserved microsynteny will be useful for further experimental functional genomic annotation.

We found that the conservation of microsynteny varied throughout the mouse genome. The average percentage of genes with conserved microsynteny in a 20 Mb interval was 38.29%, with a standard deviation of 11.81%. The Shapiro-Wilk test was applied to non-overlapping 20 Mb windows to determine whether the distribution of gene microsynteny was normal. A P-value of 0.16 indicated that the null hypothesis (a normal distribution) should not be rejected. However, the use of non-overlapping 20 Mb windows restricted the resolution of the study. For example, chromosome 19 has only two observations from non-overlapping windows. To improve the resolution of our study, we next examined 20 Mb intervals staggered by 5 Mb. This sliding window analysis allowed more observations on each chromosome.

**Figure 1:**
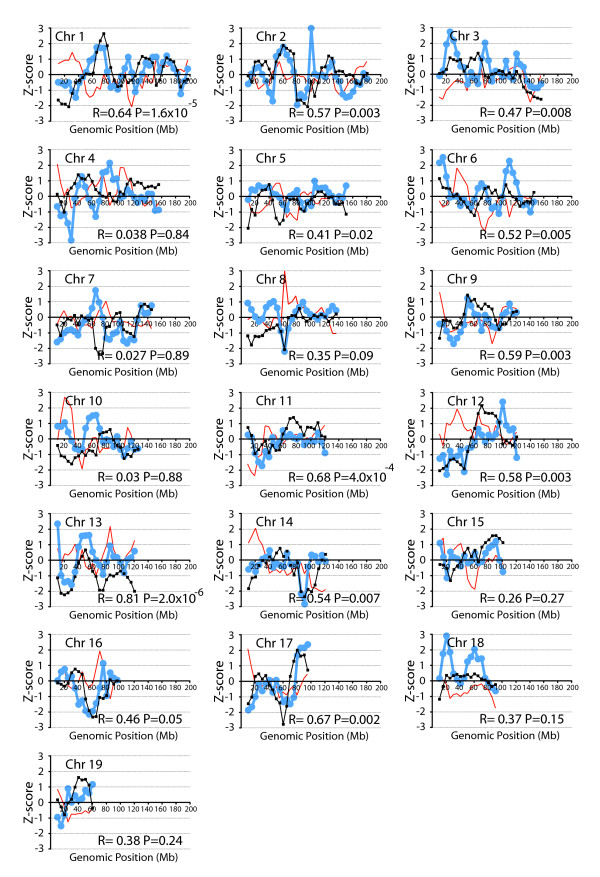
**The correlation between microsynteny and density of orthologs of disease-related genes on mouse autosomes**. The relationship between conserved microsynteny (black) and density of mouse genes orthologous to human disease-related genes (blue) is shown for all mouse autosomes. Percentage of genes with conserved microsynteny and those with disease-related orthologs are calculated for a 20 Mb sliding window, offset by steps of 5 Mb. At each position the Z-score is plotted at the center of the sliding window. Pearson's correlation coefficient and P-values for the analysis of co-localization of conserved microsyteny and disease gene orthologs are given for each chromosome. The results of a randomization of the disease genes (red) demonstrate that there is no correlation between microsynteny (black) and random assignment of gene status.

As the data conformed to a normal distribution, we therefore calculated Z-scores (number of standard deviations above or below the mean) for each 20 Mb sliding window. Windows with Z>1 were considered to have increased conservation, those with Z<-1 were considered to have decreased conservation, and windows with 1>Z>-1 had intermediate conservation. There were 51 sliding windows of the mouse genome found to have Z>1, indicative of higher microsynteny conservation, and 91 intervals found to have Z<-1, indicative of lower microsynteny conservation. Three hundred twenty-two genomic regions demonstrate intermediate microsynteny conservation with scores of 1>Z>-1. On individual chromosomes there is variation in the conservation of microsynteny, with most chromosomes containing both windows of increased microsynteny conservation and windows of decreased microsynteny conservation (Figure 1 black lines). However, mouse chromosomes 5, 7, 8, 10, 13, 14, 16 and 18 do not contain any regions of increased (Z>1) microsynteny conservation. We found that the percentage of genes with conserved microsynteny per chromosome also showed variation, with chromosome 13 having the lowest percentage of conserved genes at 25%, and chromosome 15 having the highest percentage of conserved genes at 48% (Table 1). Previous work has shown that syntenic conservation is not simply related to gene density [29].

**Table 1:** Variation of microsynteny and disease gene density on all mouse autosomes.

Chromosome	Total Genes	Conserved Genes	Percentage conserved	Disease Genes	Percentage Disease
1	1186	486	41%	98	8%

2	1846	696	38%	123	7%

3	996	379	38%	82	8%

4	1289	591	46%	95	7%

5	1219	428	35%	100	8%

6	1128	418	37%	91	8%

7	1867	650	35%	111	6%

8	1051	364	35%	89	8%

9	1219	481	39%	86	7%

10	978	321	33%	75	8%

11	1674	734	44%	122	7%

12	651	213	33%	49	8%

13	786	199	25%	66	8%

14	772	279	36%	55	7%

15	780	374	48%	65	8%

16	658	237	36%	46	7%

17	1019	369	36%	67	7%

18	495	192	39%	48	10%

19	725	318	44%	50	7%

Total for all autosomes	20339	7729	38%	1518	7%

### Comparison with sequence-based synteny blocks

We compared our results from to the sequence-based synteny blocks presented for pair-wise genome alignments on the Ensembl genome browser. For each region of the mouse genome with increased microsynteny conservation, we identified the syntenic region of the dog, rat, and human genome (Table 2, see Additional file 1). Most of the conserved mouse regions identified based on microsynteny also show conservation with a single region in the rat based on sequence. For the intervals on mouse chromosome 2 from 115 - 140 Mb, mouse chromosome 3 from 45 - 65 Mb, mouse chromosome 4 from 30 - 65 Mb, mouse chromosome 9 from 40 - 70 Mb, and mouse chromosome 15 from 65 - 103 Mb, the breakpoints of synteny in the mouse genome as compared to dog and human genome are the same, showing evolutionary conservation of genome rearrangements. In a separate study directly comparing dog and human synteny blocks, all of these regions were found to be syntenic between dog and human [41]. Although the region from 0 - 20 Mb on mouse chromosome 6 is the only region entirely conserved as a sequence-based synteny block in all three other genomes, it is not the most highly conserved region based on microsynteny in the mouse genome.

**Table 2:** Comparison of regions with Z>1 microsynteny to sequence-based synteny blocks.

Mouse Micro-synteny Interval	Dog Sequence Synteny Blocks	Rat Sequence Synteny Blocks	Human Sequence Synteny Blocks
Chr1 60 - 90 Mb	60 - 80 Mb = Chr37: 14.6 - 32.7 Mb, 80 - 90 Mb = Chr25: 40.6 - 48.1 Mb	60 - 85 Mb = Chr9: 58.5 - 84.4 Mb, 87 Mb - 90 Mb = Chr9: 84.2 - 87.0 Mb	60 - 90 Mb = Chr2: 203.6 - 234.6

Chr1 120 - 145 Mb	120 - 132 = Chr19: 31.4 - 43.3 Mb *132 - 133 Mb = Chr7: 8.7 - 9.3 Mb*, *133 - 136 Mb = Chr38: 3.0 - 5.6 Mb*, 136 - 140 Mb = Chr7: 3.0 - 7.1 Mb	120 - 145 Mb = Chr13: 30.9 - 57.8 Mb	120 - 132 Mb = Chr2: 125.6 - 138.4 Mb, *132 - 145 Mb = Chr1: 193.0 - 207.7 Mb*

Chr1 155 - 175 Mb	155 - 168 Mb = Chr7: 19.4 - 33.9 Mb, 169 - 175 Mb = Chr38: 20.0 - 25.5 Mb	155 - 175 Mb = Chr13: 68.2 - 89.3 Mb	*155 - 175 Mb = Chr1: 159.2 - 182.9 Mb*

Chr2 45 - 85 Mb	45 - 53 Mb = Chr19: 52.6 - 56.7 Mb, 54 - 84 Mb = Chr36: 3.8 - 32.4 Mb, 84 - 85 Mb = Chr18: 41.4 - 41.9 Mb	45 - 85 Mb = Chr3: 25.6 - 68.4 Mb	45 - 84 Mb = Chr2: 145.2 - 188.3 Mb, *84 - 85 Mb = Chr11: 56.9 - 57.5 Mb*

Chr2 115 - 140 Mb	115 - 127 = Chr30: 7.1 - 19.7 Mb, 127 - 129 Mb = Chr17: 37.5 - 40.1 Mb, *129 - 140 Mb = Chr24: 22.0 - 11.7 Mb*	115 - 140 Mb = Chr3: 101.6 - 128.3 Mb	115 - 126 Mb = Chr15: 36.8 - 51.2 Mb, *126 - 127 Mb = Chr2: 95.6 - 97.0 Mb*, 127 - 129 Mb = Chr2: 110.8 - 113.5 Mb, 129 - 140 Mb = Chr20: 1.4 - 13.8 Mb

Chr3 15 - 35 Mb	15 - 16 Mb = Chr24: 22.4 - 22.5 Mb, 16 - 19 Mb = Chr29: 16.2 - 18.7 Mb, *19 - 20 Mb = Chr23: 46.7 - 47.0 Mb* *21 - 30 Mb = Chr34: 43.5 - 37.0 Mb* 31 - 35 Mb = Chr34: 15.5 - 18.8 Mb	15 - 35 Mb = Chr2: 87.0 - 122.2 Mb	15 - 16 Mb = Chr20: 1.5 - 1.6 Mb, 16 - 19 Mb = Chr8: 64.0 - 67.0 Mb, *19 - 20 Mb = Chr3: 148.4 - 148.8 Mb*, 20 - 35 Mb = Chr3: 176.7 - 182.5 Mb

Chr3 45 - 65 Mb	*45 - 51 Mb = Chr19: 5.8 - 11.6 Mb*, 52 - 55 Mb = Chr25: 3.5 - 8.0 Mb, 57 - 65 Mb = Chr23: 47.2 - 53.1 Mb	45 - 65 Mb = Chr2: 132.8 - 155.1 Mb	45 - 52 Mb = Chr4: 134.0 - 140.8 Mb, *52 - 55 Mb = Chr13: 36.0 - 41.1 Mb*, 57 - 65 Mb = Chr3: 149.0 - 156.2 Mb

Chr3 55 - 75 Mb	55 - 57 Mb = Chr25: 7.6 - 8.0 Mb, 57 - 67 Mb = Chr23: 47.2 - 55.0 Mb, 67 - 75 Mb = Chr34: 28.0 - 35.3 Mb	55 - 75 Mb = Chr2: 144.4 - 166.1 Mb	*55 - 57 Mb = Chr13: 36.0 - 36.3 Mb*, 57 - 75 Mb = Chr3: 149.0 - 167.1 Mb

Chr4 30 - 65 Mb	30 - 35 Mb = Chr12: 52.6 - 49.6 Mb, 35 - 65 Mb = Chr11: 48.5 - 73.2 Mb	30 - 65 Mb = Chr5: 48.2 - 82.0 Mb	*30 - 34 Mb = Chr6: 87.8 - 93.9 Mb*, 34 - 45 Mb = Chr9: 27.3 - 38.4 Mb, 46 - 65 Mb = Chr9: 100.1 - 119.1 Mb

Chr4 105 - 125 Mb	105 - 107 Mb = Chr5: 57.1 - 58.9 Mb, 107 - 118 Mb = Chr15: 11.6 - 19.9 Mb, 118 - 125 Mb = Chr15: 3.2 - 7.9 Mb	105 - 125 Mb = Chr5: 127.3 - 144.8 Mb	*105 - 125 Mb = Chr1: 37.2 - 55.5 Mb*

Chr6 0 - 20 Mb	0 - 13 Mb = Chr14: 21.6 - 30.2 Mb 13 - 20 Mb - Chr14: 55.2 - 55.9 Mb	0 - 20 Mb = Chr4: 28.1 - 44.7 Mb	0 - 20 Mb = Chr7: 92.7 - 117.8 Mb

Chr9 40 - 70 Mb	40 - 54 Mb = Chr5: 13.7 - 27.7 Mb, 54 - 70 Mb = Chr30: 19.8 - 27.0 Mb	40 - 70 Mb = Chr8: 43.2 - 74.6 Mb	*40 - 54 Mb = Chr11: 107.4 - 123.4 Mb*, *54 - 70 Mb = Chr15: 59.4 - 78.3 Mb*

Chr11 60 - 90 Mb	*60 - 72 Mb = Chr5: 33.1 - 44.6 Mb*, *72 - 90 Mb = Chr9: 33.3 - 51.0 Mb*	60 - 90 Mb = Chr10: 46.4 - 78.6 Mb	*60 - 76 Mb = Chr17: 1.1 - 17.7 Mb*, 76 - 90 Mb = Chr17: 28.8 - 53.4 Mb

Chr11 100 - 120 Mb	*100 - 120 Mb = Chr9: 3.6 - 24.5 Mb*	100 - 120 Mb = Chr10: 89.0 - 109.7 Mb	100 - 104 Mb = Chr17: 39.6 - 45.2 Mb, 104 - 105 Mb = Chr7: 128.0 - 128.1 Mb, 106 - 120 Mb = Chr17: 60.5 - 79.2 Mb

Chr12 55 - 105 Mb	55 - 72 Mb = Chr8: 15.7 - 31.0 Mb, 72 - 102 Mb = Chr8: 36.6 - 65.4 Mb, 102 - 104 Mb = Chr8: 3.9 - 5.1 Mb, 104 - 105 Mb = Chr8: 65.4 - 66.4 Mb	55 - 105 Mb = Chr6: 74.3 - 127.9 Mb	55 - 72 Mb = Chr14: 34.2 - 52.3 Mb, 72 - 105 Mb = Chr14: 58.7 - 94.8 Mb

Chr15 65 - 104 Mb	65 - 76 Mb = Chr13: 31.6 - 41.2 Mb, *77 - 89 Mb = Chr10: 19.5 - 31.6 Mb*, *89 - 104 Mb = Chr27: 3.8 - 18.6 Mb*	65 - 104 Mb = Chr7: 103.1 - 142.6 Mb	65 - 76 Mb = Chr8: 132.9 - 146.2 Mb 77 - 89 Mb = Chr22: 36.0 - 51.2 Mb 89 - 104 Mb = Chr12: 33.5 - 54.9 Mb

Chr17 65 - 95 Mb	*65 - 66 Mb = Chr3: 4.6 - 5.2 Mb*, *66 - 72 Mb = Chr7: 72.2 - 78.8 Mb*, 72 - 84 Mb = Chr17: 25.8 - 37.3 Mb, 84 - 90 Mb = Chr10: 48.4 - 54.8 Mb, 91 - 95 Mb = Chr7: 70.7 - 72.3 Mb	65 - 72 Mb = Chr9: 103.9 - 110.8 Mb, *72 - 77 Mb = Chr6: 17.9 - 24.1 Mb*, 77 - 93 Mb = Chr6: 0.9 - 17.8 Mb, 93 - 95 Mb = Chr9: 110.7 - 112.5 Mb	65 - 66 Mb = Chr5: 109.2 - 110.0 Mb, *66 - 71 Mb = Chr18: 2.5 - 9.8 Mb*, 72 - 93 Mb = Chr2: 29.0 - 51.2 Mb, 94 - 95 Mb = Chr18: 0.9 - 2.5 Mb

Chr19 30 - 65 Mb	30 - 34 Mb = Chr26: 38.4 - 41.9 Mb, 34 - 65 Mb = Chr28: 7.3 - 32.4 Mb	30 - 65 Mb = Chr1: 233.7 - 267.8 Mb	30 - 32 Mb = Chr10: 51.9 - 54.5 Mb, 32 - 65 Mb = Chr10: 89.2 - 121.2 Mb

Sequence-based synteny blocks were identified from the Ensembl genome browser. The intervals are listed according to positions in the mouse genome, with the positions of synteny in the other genomes listed following the "=" sign. The specific region of synteny in the other species is listed following the chromosome number and a ':'. Forward to forward alignments are show in normal text, forward to reverse alignments are shown in italics.

### Conserved genes are located next to other conserved genes

Although there is variation in the density of genes with conserved microsynteny across the genome, it is possible that this variation merely represents random variation within a normal distribution. To determine whether the genomic arrangement of genes with conserved microsynteny is random, we calculated the likelihood that a gene with syntenic conservation is found next to another gene with syntenic conservation. We then compared this to the frequency of conserved-synteny neighbors in a set of 10,000 randomized genomes. In each of the randomized genomes the number and position of genes is maintained, but the annotation of conservation is randomly shuffled.

**Figure 2:**
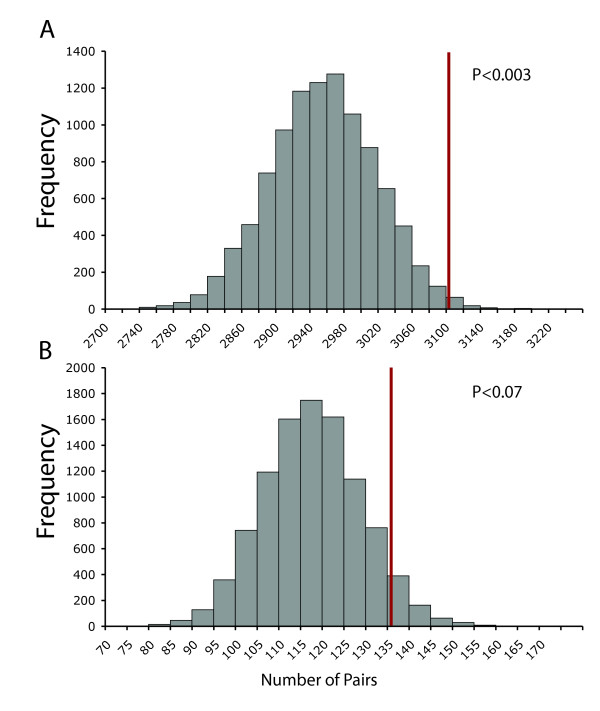
**Conserved genes and disease genes are not randomly distributed throughout the mouse genome**. Panel A: Conserved genes are found to have conserved genes as neighbors more often than expected if gene position was random. The results of 10,000 randomization trials are shown in the histogram, while the observed data (number of conserved genes with at least one conserved neighboring gene) is shown with the red line. Panel B: Disease genes neighbor other disease genes more often than expected by chance. The results of 10,000 randomization trials are shown in the histogram, while the observed data (number of disease genes with at least one disease gene as a neighbor) is shown with the red line.

We find that the frequency of co-occurrence of genes with conserved microsynteny is significantly non-random (Figure 2A, P < 0.003). By our definition, for a gene to have conserved microsynteny it must have two orthologous neighbors in all genomes examined. Thus, a pair of genes with conserved micro-synteny represents a larger block of conserved synteny. The finding that there are conserved microsynteny blocks in the genome that extend beyond groups of several genes suggests that there are constraints on genome evolution that influence gene arrangement, as the placement of conserved genes significantly differs from a random distribution.

### Distribution of mouse orthologs of human disease-related genes

As we found that conserved genes were more likely to have conserved genes as neighbors, we investigated whether any other groups of genes were found preferentially in regions of the genome with conserved microsynteny. One group of genes that are of interest is disease genes, as they are highly relevant to human health. We therefore performed a genome-wide analysis of the mouse orthologs of human disease-related genes to assess whether they were found at a greater density in conserved regions of the mouse genome. We identified human genes with a disease-associated mutant allele from the OMIM Morbid Map database, and cross-referenced them to the mouse genome using Ensembl BioMart to identify mouse orthologs. Using the genomic locations of the mouse orthologs of human disease genes from our study on microsyntenty conservation, we determined the proportion of human disease gene orthologs in each 20 Mb sliding window of the mouse genome. The mean percentage of disease-related gene orthologs as compared to the number of total genes in a 20 Mb interval is 7.68%, with a standard deviation of 2.72%. We found variation in the distribution of disease gene orthologs in the mouse genome, with the highest percentage found on chromosome 18, and the lowest on chromosome 7 (Table 1). However, on all other autosomes, 7 - 8% of the genes are orthologs of human disease genes.

### Disease gene orthlogs are located next to other disease gene orthologs

As we had found that the distribution of genes with conserved microsynteny is non-random, we examined whether that was also true for genes with disease-related orthologs. Using a similar approach, we calculated the number of mouse orthologs of human disease genes with at least one disease gene ortholog as a neighbor. As a control, we randomized which genes were annotated as disease orthologs, keeping the same total number of disease gene orthologs. From 10,000 random trails we found that the mouse orthologs of human disease genes were significantly more likely to have other disease gene orthologs as neighbors (Figure 2B, P < 0.07). This finding demonstrates that the distribution of orthologs of human disease genes in the mouse genome is not random.

### Correlation between microsynteny conservation and disease gene distribution

**Figure 3:**
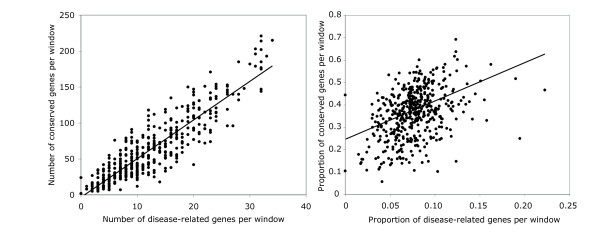
**There is a significant correlation between microsynteny and density of disease-gene orthologs over the mouse genome as a whole**. Panel A: The number of conserved genes plotted against the number of disease genes for 20 Mb sliding windows of the mouse genome. Note the fit with the regression line (Pearson's R = 0.90, P < 1 × 10^-6^). Panel B: The relationship between the proportion of genes with conserved microsynteny (number of conserved genes per window/total genes per window) and the proportion of genes with disease orthologs (number of disease-related genes/total genes per window). The correlation for the whole mouse genome is significant (Pearson's R = 0.40, P < 4.0 × 10^-4^).

We next assessed whether the orthologs of disease genes were located in regions of the mouse genome with increased microsynteny conservation. We detect a correlation between regions of the genome with conserved microsynteny and the distribution of disease gene orthologs over the whole genome (Figure 3A, Pearson's R = 0.90, P < 1 × 10^-6^). Such a representation over-estimates the true correlation between the two sets, since gene density varies considerably in different windows. This confounds the analysis as regions with high gene density would be expected to have both high numbers of disease orthologs and high numbers of genes with conserved microsynteny regardless of whether there is an additional correlation between microsynteny conservation and the presence of disease gene orthologs. When corrected for gene density, a significant correlation between microsynteny conservation and disease gene ortholog density is still observed (Figure 3B, Pearson's R = 0.40, P < 4.0 × 10^-4^).

The density of disease gene orthologs for each genomic region is shown in Figure 1 (blue lines). Z-scores are displayed to allow direct comparison between microsynteny conservation and disease orthologs. The additional calculation of Z-scores does not change the overall correlation. There is a significant correlation (P = 0.05) between microsynteny conservation and the density of disease gene orthologs for 12 of the 19 mouse autosomes. Thus, genomic regions with a high percentage of genes with conserved microsynteny also have a high percentage of disease gene orthologs. The chromosome with the best correlation between conserved microsynteny and density of disease gene orthologs is mouse chromosome 13, while the chromosome with the worst correlation is mouse chromosome 10.

To demonstrate that this correlation was not an artifact of our analysis, we randomized the annotation of disease genes. We assigned alternate genes as orthologs of human disease genes, keeping the total number of disease genes per chromosome the same as the first analysis. We then recalculated the percentage of alternate disease genes as compared to total genes in each sliding window throughout the genome, and the average and standard deviation for each sliding window. We plotted the Z-scores for each window containing these alternate disease orthologs, and compared them to the Z-scores for microsynteny conservation (Figure 1 red lines). When the chromosomal positions of orthologs of disease genes are changed to random locations, the correlation with microsynteny disappears (Pearson's R = 0.02, P < 0.58). As an additional control, we also randomized disease genes while retaining the same number of observed disease gene pairs for each chromosome. Again, we found no correlation (Pearson's R = 0.004, P < 0.93). Should the correlation between observed disease gene ortholog distribution and microsynteny conservation be an artifact of our methodology, we would also expect the randomized annotations to be correlated. This is not the case, demonstrating that the link between microsynteny correlation and density of disease gene orthologs does not arise from an artifact of the methodology.

### Robustness to changes in window size

To determine if the correlation we observed between microsynteny conservation and disease gene ortholog density was affected by the window size used in our analysis, we repeated our assays using additional window sizes. We chose to analyze window sizes of 10 Mb, 5 Mb, 2 Mb, and 1 Mb, with a stagger of one-quarter of the window size. We found that there was also a significant correlation between regions with conserved microsynteny and a high density of disease gene orthologs for window sizes of 10 Mb, 5 Mb, 2 Mb, and 1 Mb (all p < 1 × 10^-10^, Table 3, see Additional file 2). A repeat of our randomization test shows that this correlation is not significant when genes are randomly annotated for window sizes of 10 Mb, 5 Mb, and 2 Mb. However, with the small window sizes of 2 mb and 1 Mb, many genomic windows do not contain any annotated genes (Table 4), so these windows artificially show a correlation between microsynteny conservation and disease gene density, because 0 genes of either class are found in windows lacking any annotated genes. When we remove all windows with no genes from our analysis, the correlation between microsynteny conservation and disease gene density at 2 Mb and 1 Mb improves, while the randomization trial correlation loses significance (Table 3).

**Table 3:** Robustness of correlation to variations in window size.

Window size	20 Mb	10 Mb	5 Mb	2 Mb	1 Mb
**Actual annotation**	R = 0.40, p < 4 × 10^-4^	R = 0.293, p < 1 × 10^-10^	R = 0.256, p < 1 × 10^-10^	R = 0.157, p < 1 × 10^-10^	R = 0.154, p < 1 × 10^-10^

**Randomized annotation**	R = 0.02, p = 0.58	R = 0.018, p = 0.59	R = 0.058, p = 0.11	R = 0.046, p = 0.45	R = 0.035, p = 0.01

**Actual annotation, empty windows removed**				R = 0.11 p < 1 × 10^-10^	R = 0.11, p < 1 × 10^-10^

**Randomized annotation, empty windows removed**				R = 0.012, p = 0.40	R = 0.020, p = 0.83

Adjusting the window size does not eliminate the correlation between regions of conserved microsynteny and regions of high density of disease gene orthlogs. Randomization of gene annotations does eliminate the correlation at all window sizes except 1 Mb. However, when windows with no annotated genes are omitted from the analysis (bottom two rows), the correlation for actual annotations is improved at both 2 Mb and 1 Mb window sizes, and is eliminated for random annotations. As the genome does not have any windows lacking gene annotation for windows of 20 Mb, 10 Mb, or 5 Mb, the correlation values could not be recalculated to omit annotation-free windows at those window sizes. R = Pearson's R.

**Table 4:** Number of windows in mouse genome at varying window sizes.

Window Size	20 Mb	10 Mb	5 Mb	2 Mb	1 Mb
**Total windows**	465	1912	3718	4897	24541

**No annotated genes**	0	0	0	237	2761

**No conserved genes**	0	22	244	1211	9505

**No disease genes**	2	92	634	2160	14598

**No disease or conserved**	0	16	166	987	8243

The number of windows with no annotations of genes, conserved genes, or disease orthologs is shown. Note that the bottom row shows the number of windows lacking both conserved and disease genes, which are a subset of the number of windows with either no disease genes or no conserved genes.

## Discussion

We have examined the relationship between microsynteny conservation and the density of orthologs of human disease genes in the mouse genome. We found a correlation between regions of conserved microsynteny and the location of mouse orthologs of human disease genes, which is consistent for variations in the window size used in our analyses. The correlation we observe suggests that regions of the mouse genome with a high density of disease gene orthologs undergo less rearrangement than regions of the genome with fewer disease gene orthologs. Genes associated with human disease are often orthologous to essential genes in other organisms [42]. Previous studies from both mammals [29] and other eukaryotes [24,26] have shown that essential genes are located in highly conserved genomic regions. Thus, disease-related genes, which perform essential functions, are more likely to be found in conserved regions of the genome.

Several studies have found that at the sequence level, human disease genes are more conserved than non-disease genes [26,43,44]. The sequence conservation of human disease genes with essential *C. elegans *orthologs is higher than those disease genes whose orthologs are not lethal when mutated [44]. Interestingly, genes with high polymorphism among humans, but no divergence between humans and chimpanzees, are highly associated with Mendelian disease [45]. Similarly, human disease genes with weak negative selection, where mutant alleles persist in the population, are more likely to cause diseases with Mendelian inheritance [46]. Mendelian disease genes are more constrained evolutionarily than disease genes with non-Mendelian inheritance patterns [45]. Together, these observations support our finding that the mouse orthologs of human disease genes are preferentially found in genomic regions with high microsynteny conservation.

Recombination may be mutagenic due to the possibility of unequal crossing-over. Thus faulty recombination events in regions with essential genes are likely to be deleterious to the survival of the organism and may thus be selected against during mammalian evolution. Studies of the human genome support this link between low recombination and essential genes. Regions of the human genome with high linkage disequilibrium, and thus low recombination, are enriched for genes associated with essential cellular functions such as response to DNA damage, cell cycle progression, or DNA and RNA metabolism [47]. Genes that show variation in populations, such as immune response genes, are often found in regions with low linkage disequilibrium, suggesting that recombination in these regions is not deleterious to the organism [47]. Likewise, human genes found in mutation cold spots tend to be genes involved in essential cellular processes, while those in mutation hot spots include immune response genes [48]. These findings extend to non-coding sequences as well, as human genomic regions that are highly conserved with the pufferfish have been found to contain enhancers for developmental genes [49].

The correlation between disease gene density and microsynteny conservation, although significant, is not perfect. Discrepancies may come from several sources. For example, annotation of human disease genes is incomplete. Many housekeeping genes, which are likely to be essential for mammalian development, are not annotated as human disease genes, probably because mutations in these genes are lethal early in development, and thus humans with mutations are not viable [50]. The genomic region between 55 - 75 Mb on mouse chromosome 3 shows high conservation but a low density of disease gene orthologs. However, the genes *Wwtr1 *and *Shox2 *are located in this genomic region. A mouse knock-out of *Wwtr1 *displays a phenotype resembling human polycystic kidney disease [51], and the mouse knock-out of *Shox2 *is lethal with cleft palate [52], strongly suggesting that these genes are linked to human disease, although neither is annotated as a disease gene in OMIM.

Likewise, many genes that are annotated as human disease genes may not be strictly essential for survival, and thus these genes are not expected to have conserved microsynteny. The genomic region between 85 - 105 Mb on mouse chromosome 12 has a high density of disease gene orthologs but low conservation. Mutations in the human gene *SERPINA10*, whose ortholog is located in this region, are associated with susceptibility to deep vein thromboses [53]. Although *SERPINA10 *is annotated in OMIM as a disease gene, it is unlikely that inherited mutations in *SERPINA10 *present a challenge to survival of the individual, suggesting that *SERPINA10 *does not represent an essential gene. Finally, many diseases, especially cancers, are caused by translocation events that produce chimeric proteins. While a genomic region may have a great density of disease loci due to translocations, these regions would not show microsynteny conservation, as they are high in rearrangements.

Discrepancies between microsynteny conservation and the density of disease-related gene orthologs may also arise because other factors contribute to selective pressure on genome evolution. For example, previous studies have suggested that mammalian genes are clustered into groups based on co-expression [54,55]. It is proposed that gene expression is therefore an evolutionary constraint on genome organization, although the effect is weak as gene clusters are found only slightly more often than by chance [55]. There is also evidence that many over-lapping gene pairs exist in mammalian genomes, and that these gene pairs are conserved in multiple species, probably because recombination or mutation in these regions of the genome would cause deleterious mutations in both genes [56]. Alternative mechanisms for the presence of essential genes constraining genome structure have also been proposed [24].

## Conclusion

We have demonstrated the non-random distribution both of genes with conserved microsynteny and genes with disease orthologs. This observation suggests that there are constraints on genome organization in the mouse. Moreover, we have demonstrated that there is a correlation between mammalian genome architecture and gene function. It is likely that this correlation arises from gene function constraining genome organization, resulting in essential disease genes being located in regions of the mammalian genome with high conservation. The identification of a correlation between microsynteny conservation and density of disease gene orthologs suggests that additional experimental analysis of mouse genes in highly conserved genomic regions will produce new mammalian disease models by creating mutations in the orthologs of human disease genes.

## Methods

### Microsynteny conservation

All protein coding genes on mouse autosomes were retrieved using a BioMart search on the Ensembl Genome Browser (http://www.ensembl.org/index.html, release 50, NCBIM37 dataset). Orthologs of these genes in the human, rat, and dog genomes, along with their genomic positions, were retrieved using Ensembl BioMart homology filters. Any mouse genes that did not have an ortholog in another genome identified in BioMart were then subjected to a BLAT search http://genome.ucsc.edu/cgi-bin/hgBlat?command=start on the other genome using the mouse protein sequence retrieved with the BioMart sequences filter. The genomic location of the best match to the mouse protein sequence from the second genome was retrieved. Locally developed software was used to identify genes with conserved microsynteny. Each mouse gene is analyzed in turn. For each mouse gene m, its neighbors in the mouse genome (m-1 and m+1) are identified. The orientation of m-1, m and m+1 is determined from the start and stop positions. In the dog, human and rat genomes, the homologue of m is identified from both Ensembl BioMart homology filters and BLAT searches. We term these homologues d, h and r. For each of these genes their neighbors in their respective genomes (d-1, d+1, h-1, h+1, r-1 and r+1) are identified, and their orientation determined. The gene m is defined as having conserved microsynteny only if all of the following are true: (i) m, d, h, and r comprise a homologous set of genes (ii) m-1, d-1, h-1 and r-1 comprise a homologous set (iii) m+1, d+1, h+1 and r+1 comprise a homologous set (iv) the m, d, h, and r have the same orientation (v) m-1, d-1, h-1 and r-1 have the same orientation and (iv) m+1, d+1, h+1 and r+1 have the same orientation. Results are robust whether or not we limit the definition of conservation only to those genes close to each other in the genome (within 200 Kb). Results presented have conservation limited in this manner.

Once conserved microsynteny had been defined for each gene the percentage of genes with microsynteny was determined for 20 Mb intervals of the mouse genome, staggered by a 5 Mb sliding window. To define the percentage of microsynteny for a given window, the number of genes with conserved synteny was divided by the total number of genes in that window. The position of each gene was chosen as the start site listed in Ensembl. For genes with multiple transcripts, the position of the start site of the longest transcript was used. The Shapiro-Wilk test was applied to non-overlapping 20 Mb regions of the mouse genome to determine whether the distribution gene microsynteny was normal. Z-scores were calculated for each region of the mouse genome using the equation:

Where x is the proportion of genes with conserved synteny within each window, μ is the mean proportion of genes with conserved synteny in all windows and s is the standard deviation of genes with conserved synteny in all windows. Thus the Z-score represents the number of standard deviations above or below the mean.

### Comparison with sequence-based synteny blocks

Each highly conserved mouse genomic region was compared to synteny maps based on sequence alignment. Maps of the mouse compared to dog, rat, and human were retrieved from the Ensembl genome browser (Comparative Genomics - Synteny, http://www.ensembl.org/Mus_musculus/Location/Synteny) database. Genomic positions of synteny blocks were retrieved from the database and tabulated.

### Statistical analysis of conserved and disease gene pairs

Genes annotated as having conserved synteny or as disease orthologs were examined to determine whether the following gene on the chromosome was similarly annotated. The number of such gene pairs was determined per chromosome. By this definition a run of three genes with similar annotation would be counted as two pairs.

To determine the significance of the frequency of such gene pairs, we compared the observed value to a random distribution. The random distribution was calculated by keeping the both the total number of genes and the number of genes with a given annotation constant, but randomizing the assignment of those annotations over the total number of genes. The annotations were randomized 10,000 times, and the number of neighbors with similar annotations calculated. The significance of the observed value was determined from this simulation i.e. the likelihood of the observed value is the proportion of random values that are greater than or equal to the observed value.

### Disease-related ortholog distribution

Human genes with a disease-associated mutant allele were identified from the OMIM Morbid Map database http://www.ncbi.nlm.nih.gov/Omim/getmorbid.cgi. These were cross-referenced to the mouse genome using Ensembl BioMart homology filters to identify mouse orthologs. The distribution of disease-related gene orthologs was analyzed by sliding window analysis, in the same manner as for conserved synteny. For each 20 Mb window the number of disease gene orthologs was divided by the total number of genes in a window to determine the proportion of disease gene orthologs in the window. Z-scores were then calculated for each window using the equation above.

### Correlation analysis

The Pearson's correlation between the microsynteny conservation Z-scores and disease-related ortholog Z-scores was calculated, and significance calculated using the chi-squared test. As a control, we assigned new disease genes at random for each chromosome, keeping the total number of disease genes per chromosome the same. We then recalculated the density of disease genes in each sliding window throughout the genome. We plotted the Z-scores for each window containing these alternate disease orthologs, and compared them to the Z-scores for microsynteny conservation. The Pearson's correlation was then re-calculated, showing no significance for the alternate set of disease genes. Correlation analysis was also performed on window sizes of 10 Mb, staggered by 2.5 Mb, 5 Mb, staggered by 1.25 Mb, 2 Mb, staggered by 0.5 Mb, and 1 Mb, staggered by 0.25 Mb. Randomization trials were performed on 10,000 random annotations for each alternate window size as a control. To account for windows with no genes at the window sizes of 2 Mb and 1 Mb, the correlation analysis was repeated with all windows containing 0 genes removed from the actual and randomized datasets.

## Authors' contributions

SCL: performed research, analyzed data, wrote paper; XL and NRW: performed research; KEH: designed study, performed research, analyzed data, wrote paper. All authors read and approved the final manuscript.

## Supplementary Material

Additional file 1 **Gene list for regions with microsynteny conservation of Z>1**. An excel file listing all the genes (by Ensembl ID and gene symbol) found in mouse genomic regions with microsyteny conservation of Z>1. Click here for file

Additional file 2 **Data on conserved and disease genes by window for mouse genome**. An excel file listing for each window in the mouse genome the number of conserved or disease genes, with the Z-score of each window. Each window size is presented as a separate worksheet. Click here for file
